# Gossip

**Published:** 1863-10

**Authors:** 


### ART. XIII.—GOSSIP.

THE quarter has been rich in gossip. Although we have already found a place for some of the more important subjects in previous pages, we are still somewhat at a loss how best to deal with the untouched superfluity. The meeting of the British Association at Newcastle, under the presidency of Sir William Armstrcng, tempts us with its wealth of suggestion. The address of the president alone claims even an alarmed attention. It is not an easy matter to get rid of the awful picture that he drew of a possible burnt-out England, say two centuries to come. It is still less easy to forget that every domestic hearth in the kingdom is aiding as best it may in hastening our national doom. Luxuriating in our wealth of coal, we use it as lavishly as if it would last for ever, and in so acting, poison the present generation, and doom the future. Listen to what Sir William Armstrong says of our extravagant mode of employing coal for domestic purposes:—"It is computed that the consumption of coal in dwelling-houses amounts in this country to a ton per head per annum of the entire population: so that upwards of 29,000,000 tons are annually expended in Great Britain alone for domestic use. If any one will consider that 1lb. of coal applied to a well-constructed steam-engine boiler evaporates 10lb. or one gallon of water, and if he will compare this effect with the insignificant quantity of water which can be boiled off in steam by 1lb. of coal consumed in an

---

ordinary kitchen fire, he will be able to appreciate the enormous waste which takes place by the common method of burning coal for culinary purposes. The simplest arrangements to confine the heat and concentrate it upon the operation to be performed, would suffice to obviate this reprehensible waste. So also in warming houses, we consume in our open fires about five times as much coal as will produce the same heating effect when burnt in a close and properly constructed stove. Without sacrificing the luxury of a visible fire, it would be easy, by attending to the principles of radiation and convection, to render available the greater part of the heat which is now so improvidently discharged into the chimney. These are homely considerations—too much so, perhaps, for an assembly like this; but I trust that an abuse involving a useless expenditure exceeding in amount our income-tax, and capable of being rectified by attention to scientific principles, may not be deemed unworthy of the notice of some of those whom I have the honour of addressing." And so it comes to pass that cook, wielding her poker in the kitchen, stands out before the eyes of modern science as a disguised Alecto, polluting the air with pestilential vapours and hastening the extinction of peoples.

Constituted authorities as little avail against the public evil which thus arises, as household powers to control this dreaded domestic Fate. The Metropolitan Association of Medical Officers of Health are crying aloud to governing bodies to abate the smoke nuisance in London, but as yet a deaf ear is turned to their appeal. But let us not be too hasty to blame. Metropolitan sanitation is in a pitiable dilemma just now. It was priding itself upon the great works it was carrying out, and in the near prospect of the huge province of houses, and the mighty river which traverses it, being cleansed from their superabundant filth. It saw with just pride the near culmination of years of anxious thought and work, and pictured to itself an overflowing population informed with new life and vigour. But its cup of rejoicing has been infused with bitterness. An outcry has been raised against its representative, the Metropolitan Board of Works. In their blind perversity, it is said, they are casting away, not the filth of the town, but the riches of a nation. The sewage which they seek to drown in the Thames at a distant point, would regenerate a people. They are spending millions to waste millions. The land is starving for the filth they would cast so remorselessly away; and the people are starving for the rich harvests it would create. Looking into the streams of foul and stinking matter (we beg pardon, into the vital elixir) which flow deep beneath our streets, Liebig and other chemists, see visions of waste places made glad, barren hills covered with golden harvests, arid plains rich with verdure, and starving peoples rejoicing in abundance. And this Elysian dream a mere material Board of Works ruthlessly destroys—nay, more, adding insult to injury, would subject it to base commercial considerations.

With dignified modesty the Metropolitan Board of Works have again given heed to the statements and protests of these chemists. Assuming that the sewage of a great town is as useful for agricultural purposes as it is stated to be, they have advertised for plans and estimates by which its valuable properties might be secured. But never before did scientific mountain bring forth so intolerably small a mouse. Nine estimates were laid before the Board, all of which concurred in setting down the money value of the sewage at an enormous amount; but none fully complied with the terms of the advertisements, none solved the actual practical difficulties of the question, and the majority did not even treat upon them, and all showed a peculiar diffidence in offering to purchase this myriad-valued refuse! The sands of the Rhine contain much fine gold, and the bed of the ocean is lumbered with wealth, but the question is how to get at it.

Sewage and the Thames recall St. Thomas's Hospital. The association may seem a strange, but it is a legitimate one. The southern border of the Thames is to be the seat of the future St. Thomas's. The Governors have determined to purchase, and the Metropolitan Board of Works to sell, the site to be formed partly by the removal of buildings, partly by embankment of the river, at Stangate, near the south end of Westminster Bridge, and directly facing the Houses of Parliament. There the new hospital will be built. The Governors of Bethlehem have suffered to escape this great opportunity of doing honour to themselves, carrying out more completely the intention of the founders of the asylum, and giving a fuller development to the benefits to be derived by the unfortunate lunatic from the charity.

The reasons which induced the Governors finally to select this site are doubtless those submitted to them in a letter of the medical staff, on the 19th of June last. In this letter the entire staff recorded their opinions on the merits of the different sites proposed for selection. Two only, they said, were of first-rate excellence; namely, the site occupied by Bedlam, and that at Stangate. Either of these sites, they held, would be admirably suited for the reconstruction of the hospital; either of them would be incomparably superior to any third site which has been named for the purpose.

"Before arriving at this conclusion," they state, "we adverted, of course, to many different considerations—to the antecedents of the charity, to the nature of the claims which it has to meet, to the plan on which the masses of South London population are distributed, to the relative position of other hospitals, to the direction of the great streams of metropolitan traffic, to the proximity of important railway stations, and so forth. Nor, you may be sure, did we overlook the fact that higher levels of ground than the levels of Bedlam and Stangate have, *cæteris paribus*, some sanitary advantages. But, in our opinion, the pursuit of those advantages in the present case would necessarily lead to the sacrifice of more important objects; for the population which our hospital has, ever since its establishment, specially served—the population which by long usage, if not by law, has peculiar claims on the charity—is a population which, for its own purposes, has from time immemorial clustered its myriads of dwellings and work-places over a great low-lying area; and within this area the hospital must stand, unless its former usefulness to the population is to be lost. Fortunately, however, the special disadvantage of this area—the disadvantage of imperfect drainage—is one almost entirely amenable to treatment; and we are led to believe that within a few months from the present time (when the great sewerage works now in progress shall have been completed) the low-lying South London districts will not be appreciably worse drained than the higher levels of London."

Proceeding to compare with one another the two sites named, the medical staff unanimously thought that Stangate, for the proposed purpose, was superior to Bedlam, and for the following reasons:—

"Our conclusion," they say, "would, of course, have been different if we had looked forward to the river's continuing, as at present, polluted with all the refuse of this vast metropolis. But from the moment (now very soon to arrive) when new and distant outfalls shall have been provided for the refuse of London, the Thames, instead of being a nuisance to people who dwell beside it, will be a most important agent of pure ventilation; and proximity to the river—as to a vast open space which can never be built upon—will be a well-secured sanitary advantage of the very highest value. Attaching great importance to this consideration, we attach not less importance to the peculiarly commanding position which the hospital at Stangate would occupy with reference to various very large and increasing streams of London traffic; and we are of opinion that in these two respects the Stangate site is decidedly superior to Bedlam. Moreover, while we strongly support the claims which in our opinion the South London population has on the resources of St. Thomas's Hospital, we yet should regard it as an advantage, that if the hospital were at Stangate, not exclusively the South London population, but likewise a considerable and very poor section of the North London population would be within easy reach of its benefits.

"In this respect, and, indeed, in most others, the Stangate site is strikingly analogous to the very admirable old site of the hospital. The differences, such as they are—the frontage immediately to the river, the much ampler space for building, the adjacency to Westminster-bridge instead of the adjacency to London-bridge,—these, one and all, are, in our opinion, differences for the better—differences with which our future hospital may fulfil, even more thoroughly than our former hospital, the humane intentions of its founder.

"The entire medical staff of the hospital, therefore, desires respectfully to submit to the General Court of Governors its unanimous and most earnest recommendation that, unless unforeseen circumstances should render the object unattainable, the river-side site at Stangate be chosen for the reconstruction of St. Thomas's Hospital."

The recent outbreak of garotting was a somewhat rude shock to those pleasant dreams which the public have of late been accustomed to indulge in on the influence exercised upon criminals by a so-called reformatory discipline. The little effect, also, which appeared to be produced upon petty offenders by imprisonment, judging by the records of the police courts, was far from satisfactory. The entire subject of convict and gaol discipline has been submitted to official investigation, and there seems to be little doubt that in our anxiety to reform criminals, actual punishment (in the common-sense signification of the term), as a deterrant and reformatory agency, would appear to have very generally fallen into disuse. In a memorandum attached to the Report of the Royal Commission on Convict Discipline, Lord Chief Justice Cockburn observes:—

"In this country the amount of labour required from a convict under penal servitude is about one-half of what would be done by an ordinary day labourer; his diet is little, if at all, inferior to that of the ordinary labourer; he becomes entitled, on performance of the work required of him, to gratuities which by the time his term expires amount to a substantial sum; and, unless he misconducts himself, he obtains a remission of a fixed portion of the term of his sentence, varying from one-sixth on sentences of three years to one-third on sentences of fifteen years and upwards. . . . . Moderate labour, ample diet, substantial gratuities, with the remission of a fixed portion of the sentence, are hardly calculated to produce on the mind of the criminal that salutary dread of the recurrence of the punishment which may be the means of deterring him, and through his example others, from the commission of crime."

Speaking of the dietary of English convict prisons, the Governor of the Prison at Edinburgh stated (who remarked, by the way, that he had only found two prisoners who appeared to dread coming back to penal servitude) :—

"I know, not only from the statements of returned convicts and the published dietary tables, but from having personally examined the dietary in three of the principal convict prisons in England, that it is not only much superior to that of the Scotch county prisons and poorhouses, but, what is of much greater and graver importance, it is far superior to that which the honest labouring man or mechanic having a family to support can possibly procure. *The struggle for bread is the main struggle of life, and no punishment will ever appear very formidable to the poor where for comparatively light labour there is good and warm clothing, excellent lodging, and abundant food.*"

The Report of the Select Committee of the House of Lords also shows in gaols and houses of correction a weakening of the penal element of punishment, and also too frequently an extravagancy of dietary. The Committee class industrial occupation under the head of light labour; and they conclude that —"whilst industrial occupation should in certain stages form a part of prison discipline, the more strictly penal element of that discipline is the chief means of exercising a deterrent influence, and therefore ought not to be weakened, as it has been in some gaols, still less to be entirely withdrawn."

It would appear, indeed, to be evident that in our attempts to escape from the older barbaric and brutalizing system of penal discipline, we have run into the opposite extreme, and that the punishment allotted in gaols to criminals is too little painful or irksome to exercise an eminently deterring influence. The Select Committee advise a systematic adoption of the old forms of hard labour again—the tread-wheel, the crank, and the shot-drill—under definite regulations, to be made common to all prisons. They would apply to all offenders undergoing a short sentence, or are working out the earlier stages of their imprisonment, unless the prisoner is exempted by medical authority on account of ill health. The industrial occupation they would restrict to the later periods of imprisonment of those criminals who are not confined for short terms. "On all grounds," they say, "it ought to follow upon the hard labour of the tread-wheel or crank, and ought not to be accepted as an equivalent for them."

But, in considering the question of hard labour, the Committee lay down (1), that the health of the prisoner must be sustained under the discipline imposed; (2) that the labour should be administered in the most economical form possible; and (3) that due punishment should be inflicted.

In reference to these propositions, Dr. Edward Smith submitted to the Committee certain opinions on the dietary of prisoners, which bid fair to make the solution of this question the clue to the establishment of a system of penal discipline on sound scientific principles.

"It would seem to me," he said, in his evidence before the Committee, "that the right course of proceeding would be this, to determine the amount of food which is necessary to maintain a person in fair health in the open air, and to endeavour so to arrange that it shall also maintain the prisoner in health in a state of confinement. The difference of the two conditions is mainly, or perhaps entirely this, that in confinement you have less vital action in the body, less digestion of food, and less assimilation of food or conversion of food into the tissues of the body. The aim, therefore, should be so to arrange the prison discipline that there shall be an increase of this assimilation over the present amount with inaction as shall enable the cheap food which is sufficient for the support of an agricultural labourer to keep the prisoner in health. If that be not done, it will be necessary, as we do at present, to give more nitrogen. With the deficient assimilation existing in confinement, you must increase the meat or the milk—the nitrogenous foods—in order to increase the vital action of the body; but if you adopt the other course, that of giving them exercise and fresh air, such as a labourer would have, you do not need to give a proportionate increase of nitrogen; you therefore assimilate the conditions of a prisoner much more to those of an ordinary labourer, and you will not give them a dietary beyond that of any ordinary labourer, either in quantity or in quality."

From prisons and prisoners to the law-courts is a natural step. The judicial proceedings of the quarter record several cases of murder, or attempted murder, by lunatics. We briefly quote the more remarkable examples:—

A woman, aged 36 years, the mother of five children, drowned two of her children and herself. The family were in great destitution, and the husband infirm. She had several times threatened to drown herself, and on one occasion told her husband that he could support two children, but not five, and that she should destroy herself and the three young ones, of whom she was devotedly fond.

A formidable attack was made by a lunatic with a knife upon two passengers in an express train from Liverpool to London. A life-and-death struggle was maintained over a distance of forty miles. The lunatic acted under the delusion that his companions were thieves, planning to rob him.

A man was tried before Mr. Justice Willes, at Dorchester, on the 25th July, for the murder of his son. The evidence of insanity at the time of the act was complete, and the prisoner was acquitted on that ground.

A female was tried before Mr. Justice Willes at Exeter, on the 28th July, for the murder of her infant. The prisoner was a married woman, and had four children. The brother of the prisoner testified as follows :—

I went into the prisoner's room before six o'clock that morning. The husband was in bed asleep. I saw the baby on the rug. I looked at it, and saw that its throat was cut. My sister was not there. I went to the door, and saw my sister coming upstairs with a pitcher of water. I said, "Oh, Elizabeth, what have you done to the baby?" She looked at me very innocently, and said, "I have cut its throat." She went into the room and sat down on a chair, and seemed like some one lost. I said, "Oh, Elizabeth, what did you do this for?" She said, "I don't know." I said, "What did you do it with?" She said, "A razor;" and she reached it from the table. She said she had been working hard all night, and thought she should kill all in the house. I then went for a surgeon. When I came back the child was just dying. The prisoner said, "The Lord had forsaken me, and the Devil had gained the mastery over me. Oh, why did he order me to wean the child? I tried to do it two or three times. I got out of bed in the night, and first I thought I would do it, and then I thought I would not do it; but some one said 'She must do it.' I thought I was going to die, and that the child had better die first." I remained, and she told me to put everything out of her reach, for fear she might kill some one else. Some time before this she said she thought she and her husband were going to die, and that the children would come to the workhouse.—Acquitted on the ground of *insanity*.

A silk-weaver, living in Bethnal-green, murdered his wife while in a state of lunacy, and then committed suicide.

A married woman attempted to murder her eldest daughter, aged ten years, by cutting her throat, and then hung herself.

These cases do not, unfortunately, exhaust the catalogue. It might be thought that the frequent recurrence of horrible murders, in which the question of lunacy is raised, would have induced our legal authorities so far to modify the course of procedure in those instances where a doubt rests upon the sanity of the accused, as to permit the question to be dealt with as a simple matter of "fact," as the Lord-Chancellor would have it dealt with. It seems, however, that we are still to submit to the occasional exhibition of the hideous farce of a probable lunatic being sentenced to death, because a form of procedure, which the judge is competent to set aside, is maintained, under circumstances where its exercise is fatal to justice. Or we are still to suffer from time to time painful fears that a lunatic may have been consigned to the gallows because no provision is made for the careful determination of the mental state of a prisoner, upon whose competency doubt rests, and whose friends have not the means to obtain the evidence requisite to solve the question. We long ago suggested that it would be well in such cases for the judge to adjourn the case, rather than permit it to be decided upon hurried observation. The prisoner suffers nothing by the adjournment (since, if acquitted on the ground of insanity, he is not entitled to be set at liberty), and justice would be a gainer by it.

The following case, tried at Maidstone before Mr. Baron Channell, on the 15th July, is another illustration of the necessity of a change such as that we have advocated.

Alfred Holden, a soldier of the Royal Artillery, was indicted for the wilful murder of his infant son at Brompton, in this county.

There was no doubt whatever as to the prisoner's commission of the dreadful act. The prisoner was married, and had only this child, an infant about eleven months old. He had been employed as cook in the sergeants' mess at Brompton, and on the last day of March last had been discharged from this employment, or resigned it, in consequence of a few words with one of the sergeants, and on the day in question (Wednesday, the 1st of April), was to go to his regular duty. About seven o'clock on the morning of the 1st of April last a female neighbour, being sent for, went into the prisoner's room, where he and his wife were, and on the bed saw the body of the infant weltering in its blood. She said to him, "Holden, why did you do it?" And he said, "I did it; I want to be hung. I want to leave no burden on my wife; she can do for herself." The child's body was then quite warm. The witness's husband soon afterwards said to the prisoner, "Is it a fact that you are guilty of this deed?" The prisoner took him by the two hands, and said, "Yes, I am. I've done it, and I will hang, and leave no burden on her." Under the bed was a knife, which the wife pointed out to the witness. The prisoner appeared to be sober at the time, and seemed to know what he was saying, but he had been drinking the day before. He on the same morning, and very near the same time, said to another witness, "It is out of no spite to my wife; it is all through a sergeant of ours. He has tormented me for many months, and tried to injure me all he could and to get me out of my place, and, more than that, he has spoken wicked things against my wife, and I could not stand it." The witness said to him, "Why, then, did you not leave the place?" And he said, "If I had done so she could not support the child, so I thought I had better take the child's life than another's, as he had no soul to save." The corporal who arrested him, and to whom he said, "I did it," asked him his reason for doing it, to which he replied, that he wanted to get rid of the world. The corporal said if that was what he wanted, why did he not make away with himself? He replied that, "if he had done so he should leave his child a burden on his wife, whereas if he murdered the child he should then be hung, and his wife would only have herself to keep." On his way to the station, he made a rambling statement that he had been very much grieved by a sergeant belonging to the mess, and that it was this which caused him to commit the deed. To a policeman who took him into custody, he said he had killed the child, and that it was through a sergeant who had tried, he said, to do him all the harm he could. To the police superintendent, when in custody, he was stated to have said, "This is a case of spite. I did this through a sergeant of ours. I have been engaged in the mess, and he has been down upon me for some time, and it was through him I did it. I was discharged from the mess yesterday, but he did not discharge me. I discharged myself." Before the magistrates he did not deny the fact that he had committed the dreadful act.

Mr. Barrow and Mr. Marsham conducted the case as counsel for prosecution.

No one appeared as counsel for the prisoner.

On being arraigned he pleaded "Guilty."

Mr. Avory, the Clerk of Arraigns, explained the matter to him, but he persisted in his plea.

The learned Judge then explained the nature of the charge, and assured him that his confession would make no difference in his punishment.

He still, however, with every appearance of intelligence, persisted in his plea of "Guilty."

The plea was thereupon recorded, and the prisoner was removed from the bar.

The learned Judge in the meantime made certain inquiries as to the prisoner's state of mind, and soon afterwards directed him to be replaced at the bar, and he was called upon in the usual way to say if he had anything to urge why sentence of death should not be passed upon him.

The prisoner, in a tone of perfect intelligence, but indifference, said "Nothing."

The learned Judge then put on the black cap and proceeded to pass sentence as follows :—"You have been committed on your own confession of wilful murder. The crime of murder is one of the greatest known to the law, and in your case the person you murdered was your own child—an infant of less than a year in age. I am willing to believe that you were not influenced by those feelings of malice and revenge which sometimes instigate persons who commit the crime of murder. The excuse you have made for yourself, as I understand the depositions, is that you had so misconducted yourself as cook of a regimental mess that you found yourself obliged to leave the neighbourhood, and that you destroyed your own child that it might not be a burden on your wife. I cannot allow that excuse to interfere with the sentence I am called upon to pronounce. You have pleaded 'Guilty,' after being repeatedly advised, and learnt that it would make no difference in your sentence or in the punishment you had to undergo. I have felt it my duty to make every inquiry I could as to what had been observed of your conduct while in custody, but I have ascertained nothing which can lead me to believe that you were not responsible for your actions, or that you were not so at the time you committed the offence. Under these circumstances my duty is to pass upon you the sentence of the law. There my duty ends." (The learned judge then passed sentence of death in the usual form.)

The prisoner listened without apparent emotion, and retired from the bar.

Is a gossip during the sitting of the Court (for the inquiries which the Judge would then have the opportunity of making cannot well be dignified with a higher designation) to be considered as a fitting or likely means of determining a question which involves the issue of life or death? Is it conducive to confidence in the administration of justice, in these sad cases, that the incoherent statements of an undefended prisoner, who persisted in pleading "Guilty," and which statements gave rise in the Judge's mind to a suspicion of mental incompetency, should be referred to as if they were rational attempts of a man seeking to palliate his admitted offence, and deprecate the extreme rigour of the law? Finally, is it seemly that the Bench should pass the extreme sentence of the law under circumstances which must necessarily lead to an immediate and specific inquiry as to the propriety of the sentence being carried into execution?

